# Potential for direct interspecies electron transfer in an electric-anaerobic system to increase methane production from sludge digestion

**DOI:** 10.1038/srep11094

**Published:** 2015-06-09

**Authors:** Zhiqiang Zhao, Yaobin Zhang, Liying Wang, Xie Quan

**Affiliations:** 1 Key Laboratory of Industrial Ecology and Environmental Engineering (Dalian University of Technology), Ministry of Education, School of Environmental Science and Technology, Dalian University of Technology, Dalian 116024, China; 2 Department of Microbiology, University of Massachusetts, Amherst, MA 01003-9298, USA; 3 State Key Laboratory of Bioreactor Engineering and Institute of Applied Chemistry, East China University of Science and Technology, Shanghai, P.R. China

## Abstract

Direct interspecies electron transfer (DIET) between *Geobacter* species and *Methanosaeta* species is an alternative to interspecies hydrogen transfer (IHT) in anaerobic digester, which however has not been established in anaerobic sludge digestion as well as in bioelectrochemical systems yet. In this study, it was found that over 50% of methane production of an electric-anaerobic sludge digester was resulted from unknown pathway. Pyrosequencing analysis revealed that *Geobacter* species were significantly enriched with electrodes. Fluorescence *in situ* hybridization (FISH) further confirmed that the dominant *Geobacter* species enriched belonged to *Geobacter metallireducens*. Together with *Methanosaeta* species prevailing in the microbial communities, the direct electron exchange between *Geobacter* species and *Methanosaeta* species might be an important reason for the “unknown” increase of methane production. Conductivity of the sludge in this electric-anaerobic digester was about 30% higher than that of the sludge in a control digester without electrodes. This study not only revealed for the first time that DIET might be the important mechanism on the methanogenesis of bioelectrochemical system, but also provided a new method to enhance DIET by means of bioelectric enrichment of *Geobacter* species.

Anaerobic methanogenesis is an effective way to realize energy recovery from wastes^1^,^2^,^3^. Although this technology has been available for more than 60 years, it is not as widely utilized for solid waste conversion as might be expected. This is due, at least in part, to the widespread belief that anaerobic digestion is a slow process^4^. For the last decades, the working model for syntrophs and methanogens exchange electrons is regarded as interspecies hydrogen transfer (IHT)^5^,^6^,^7^. H_2_ is produced from non-methanogenic microorganisms metabolizing the fermentation products and consumed by H_2_-utilizing methanogens with the reduction of CO_2_ to CH_4_. This syntrophic metabolism of fermentation intermediates functions well as long as H_2_-utilizing methanogens maintain the concentration of H_2_ low enough that the production of H_2_ is thermodynamically favorable. Formate is an alternative to H_2_ and can also act as an electron carrier between syntrophic partners^7^,^8^,^9^. The exchange of H_2_ between the syntrophs and methanogens is a weak link. Any slight disruption in the rate of H_2_ consumption will break the balance of syntrophic metabolism, resulting in the accumulative short-chain fatty acids (SCFAs), which further inhibits the activity of H_2_-consuming methanogens to exacerbate the digester function.

Extracellular electrons are also exchanged via direct interspecies electron transfer (DIET), which is first documented in defined co-cultures of *Geobacter metallireducens* and *Geobacter sulfurreducens*^10^. *G. metallireducens* can metabolize ethanol, but cannot use fumarate as an electron acceptor^11^, whereas *G. sulfurreducens* can reduce fumarate, but cannot metabolize ethanol^12^. By DIET, *G. metallireducens* and *G. sulfurreducens* could grow in a medium with ethanol as the electron donor and fumarate as the electron acceptor. Morita *et al.*^13^ reported that the potential for direct electron exchange between *Geobacter* species and *Methanosaeta* species could happen in the brewery wastewater digesters for methane production. *Methanosaeta* species accounted for about 90% of the methanogenic archaea 16S rRNA gene sequences recovered, and H_2_-utilizing methanogens only accounted for less than 0.6% of the methanogenic archaea 16S rRNA gene sequences recovered, which implied that IHT had only a little contribution to the whole methane production^7^,^13^. [^14^C]-bicarbonate analysis suggested that DIET between *Geobacter* species and *Methanosaetae* species contributed 1/3 of methane production^7^. This discovery that *Geobacter* species transferred electrons to *Methanosaeta* species via DIET has challenged the long-held assumption that H_2_ are the primary interspecies electron carrier in conversion of organic matter into methane.

Commonly, *Methanosaeta* species are the predominant microbes in most of anaerobic methanogenic environments or anaerobic waste digesters, and the precursor of more than half of methane production^14^. However, *Geobacter* species are only frequently abundant in some limited anaerobic methanogenic environments, such as soils and sediments^15^,^16^,^17^. For some important methanogenic environments, such as anaerobic digestion of municipal sludge or of saccharides, the relative abundance of *Geobacter* species detected are low^18^,^19^,^20^. It meant that DIET from *Geobacter* species to *Methanosaet a* species for methane production was weak in these anaerobic system.

It was reported that *Geobacter* species usually adapt to grow with Fe (III) oxides^21^,^22^,^23^ or electrodes^24^,^25^ as electron acceptors. This discovery revealed the reason why *Geobacter* species could be detected in most bioelectrochemical systems with over 30–40% of 16S rRNA gene sequences recovered in the anodic microbial communities^26^,^27^,^28^. This finding predicted that the additional bioelectrochemical system might create a favorable condition to support the growth of *Geobacter* species^24^,^29^,^30^. We hereby assumed that a pair of electrodes inserted into an anaerobic digester was likely to enrich *Geobacter* species, which was expected to increase methane production via potential DIET between *Geobacter* species and *Methanosaeta* species. In this study, a single-chamber bioelectrochemical system was operated to treat waste activated sludge (WAS) with the aim to clarify the potential DIET for methane production during sludge digestion. The WAS used as the substrate was because *Geobacter* species were rare in the waste activated sludge which provided the possibility to better observe the enrichment of *Geobacter* species and its effects on methane production via DIET.

## Results

### Potential of DIET for methane production

**Figure 1:**
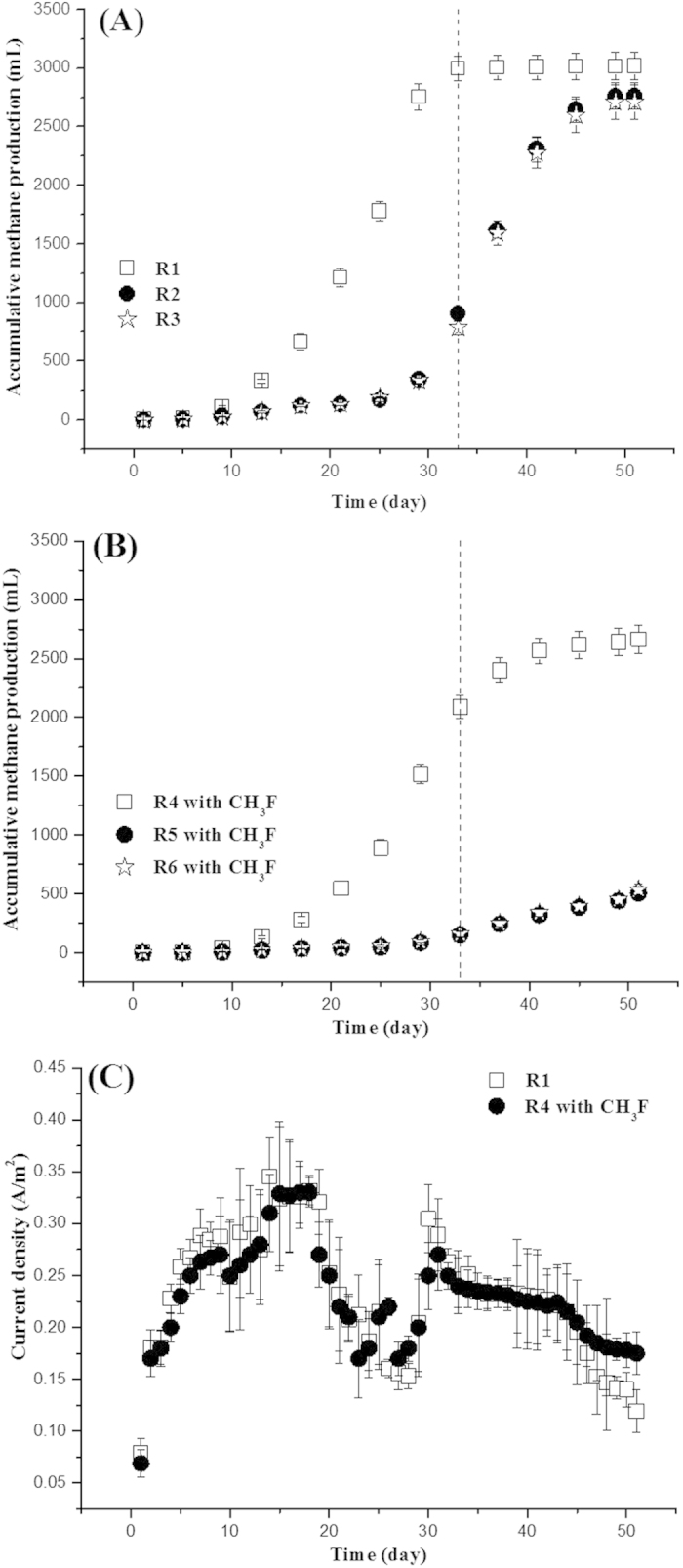
Accumulative methane production of R1, R2 and R3 (**A**) and R4, R5 and R6 with addition of CH3F (**B**). Change of current density in R1 and R4 (**C**). Error bars represent standard deviations of three groups of parallel experiments.

The accumulative methane production during 51 days experiments is shown in Fig. 1A,B. From this figure, the accumulative methane production of the bioelectrochemical system with applied voltage of 0.6  V (R1) reached 2998.4 ± 89.2 mL (mean ± standard deviation) during the initial 33 days, roughly equal to its final methane production at day 51 (3017.6 ± 107.9 mL). Comparatively, the accumulative methane production of R2 (without electrodes) was only 904.5 ± 45.5 mL at day 33, about 3/10 (or 30.2%) of that of R1. After the 51 days experiments, the accumulative methane production of R2 increased to 2763.5 ± 102.5 mL, as similar as that of R1 at day 33 (2998.4 ± 89.2 mL). Remarkably, there was no significant difference of accumulative methane production between R2 and R3 (with electrodes but without power supply) during the 51 days experiments (P > 0.05), which implied that the electrode materials themselves nearly had no effect on the methane production. These results indicated that the bioelectrochemical system had a significant contribution to the methane production. Similarly, Sasaki *et al.*^19^ had operated a cylindrical bioelectrochemical reactor using carbon fiber fabric as electrode and also obtained the enhancement of methane fermentation from thickened sewage sludge.

To assess the contribution of bioelectrochemical system to methanogenesis, the change of current density in R1 was recorded during the 51 days experiments (Fig. 1C). It is believed that exoelectrogenic bacteria like *Geobacter* species in the anodic biofilm are able to transfer the electrons from anodic oxidation of organic matters to electrode, and then hydrogenotrophic methanogens (often *Methanobacterium* species) as biocathode accept these electrons and reduce CO_2_ into CH_4_ according to the reaction: CO_2_ + 8H^+^ + 8e^−^ = CH_4_ + 2H_2_O^31^,^32^. Theoretically, the electrons might be also recovered in the cathode for hydrogen production^33^. The hydrogen production detected in R1 was only less than 0.1% (<5 mL) of total biogas production during the whole experiments, which could be neglected. The low hydrogen production was likely because ‘electrohydrogenesis’ usually required the precious metal catalysts as catalyst^34^. During the initial 33 days, the total available electrons of R1 for the reduction of CO_2_ to CH_4_ was 0.13 mol calculated by the formula (1) base on current density (Fig. 1C). Even if these electrons were totally used for cathodic methanogenese, the methane production during the initial 33 days would not exceed 403.7 mL (403.7 mL = 0.13 moL / 8 × 24.8 × 10^3^ mL/mol [the molar volume of gas at room temperature]), which only accounted for 13.5% (13.5% = 403.7 mL / 2998.4 mL × 100%) of total methane production of R1. It indicated that the combination of anodic oxidation and cathodic reduction of CO_2_ into CH_4_ was not an important mechanism for the increased methane production in R1. Thus, at least 56.4% (56.4% = [2998.4 mL − 904.5 mL − 403.7 mL] / 2998.4 mL × 100%) of the methane production of R1 should be produced from the other pathway.

Commonly, *Methanosaeta* species is responsible for directly converting acetate to methane. The acetate-utilizing methanogens are the prevailing species for methanogenesis. CH_3_F (3% [v/v], 99%) as a selective inhibitor of aceticlastic methanogenesis were added into another three parallel reactors (R4 same as R1, R5 same as R2 and R6 same as R3) to further observe the unknown methanogenesis (shown in Fig. 1B). The accumulative methane production in the three reactors all decreased with the addition of CH_3_F as compared with that of R1, R2 and R3. The accumulative methane production of R5 was only 150.9 ± 16.8  mL during the initial 33 days, which was still similar to that of R6. The methane production of these two reactors (R5 and R6) was mainly ascribed to H_2_-utilizing methanogenesis since aceticlastic methanogenesis was inhibited by CH_3_F. The accumulative methane production of R4 was 2089.0 ± 98.5 mL, significantly higher than that of those two reactors (R5 and R6). The available electrons for cathodic methanogenesis of R4 was 0.12 mol during the initial 33 days calculated by the formula (1) according to the current density (Fig. 1C). If these electrons were totally utilized for cathodic reduction of CO_2_, the accumulative methane production calculated would not excess 374.5 mL (374.5 mL = 0.12 moL / 8 × 24.8 × 10^3^ mL/mol). Thus, the unknown methane production contributed at least 1563.6 mL (1563.6 mL = 2089.0 mL − 374.5 mL − 150.9 mL) of the total methane production in R4 during the initial 33 days, which was equal to 52.1% (52.1% = 1563.6 mL / 2998.4 mL × 100%) of total methane production in R1. This methane production (1563.6 mL) was well in agreement with the unknown methane production of R1 mentioned-above (1691.1 mL, [1691.1 mL = 56.4% × 2998.4 mL]).

Considering that *Geobacter* species could directly exchange electron with *Methanosaeta* species via DIET providing another methanogenic pathway to produce methane, it was assumed that DIET might be a reason for the increased methane production in this bioelectrochemical system. If so, why the other two no-electricity reactors (R2 and R3) did not have DIET? To address this question, the microbial communities in the different reactors were analyzed.

### Analysis of microbial community structure

**Figure 2:**
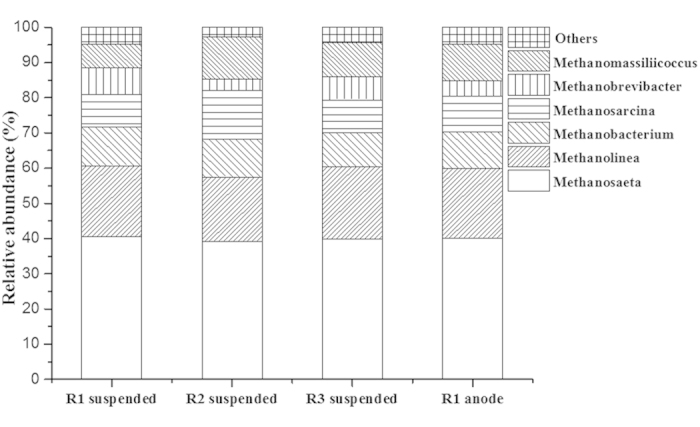
Archaeal community structure at genus level of the suspended sludge of R1, R2 and R3 and anodic biofilm of R1 based on high-throughput 16S rRNA pyrosequencing. Genera with relative abundance lower than 1.00% was classified into 32 group ‘Others’.

Archaeal microbial community structure after the 51 days experiments (Fig. 2) revealed that the most dominant genus of methanogenic archaea was *Methanosaeta* accounting for 40.55%, 39.13%, 39.82% and 40.22% of archaea 16S rRNA gene sequences recovered in the suspended sludge of R1, R2 and R3 and anodic biofilm of R1, respectively. *Methanobacterium* only accounted for 10.89%, 10.65%, 9.66% and 10.38% of archaea 16S rRNA gene sequences recovered in the above four communities, respectively. The OTUs analysis **(Fig. S1)** also showed no significant difference among the different communities.

**Figure 3:**
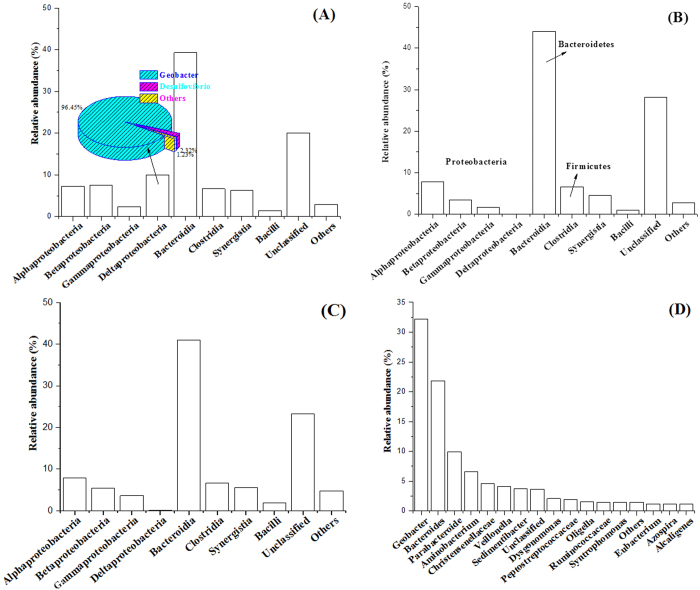
Bacterial community structure at class level in the suspended sludge of R1 (**A**), R2 (B) and R3 (C) and at genus level of anodic biofilm of R1 (D) based on high-throughput 16S rRNA pyrosequencing. Classes and genera with relative abundance lower than 1.00% was classified into group ‘Others’.

The class level identification of bacterial community structure in R1, R2 and R3 are illustrated in Fig. 3. Remarkably, *Deltaproteobacteria* species, mainly containing *Geobacter* species, accounted for 10.01% of the bacteria 16S rRNA gene sequences recovered in the suspended sludge of R1. Further identification at genus level showed that *Geobacter* species accounted for 96.45% of total sequences recovered of *Deltaproteobacteria* species in R1 (Fig. 3A). It meant that *Geobacter* species made up about 10% of bacteria 16S rRNA gene sequences in the suspended sludge of R1. Conversely, *Deltaproteobacteria* species was almost undetected in the suspended sludge of R2 and R3 (Fig. 3B,C). It indicated that *Geobacter* species was quite scarce during the anaerobic sludge digestion, making it difficult to directly exchange electrons with *Methanosaeta* species for methane production. It might be the reason for the lower methane production in R2 and R3. However, *Geobacter* species could be significantly enriched in the bioelectrochemical system based on the comparison above. It could be further confirmed by analyzing *Geobacter* species in the anodic biofilm of R1 (Fig. 3D), which accounted for 32.18% of the bacteria 16S rRNA gene sequences recovered in the anodic biofilm, significantly higher than that in the suspended sludge of R1. Based on the enrichment of *Geobacter* species and the dominant *Methanosaeta* species in the anodic biofilm and suspended sludge of R1, it was concluded that the potential DIET between these two species might be an important reason for the increased methane production in R1.

**Figure 4:**
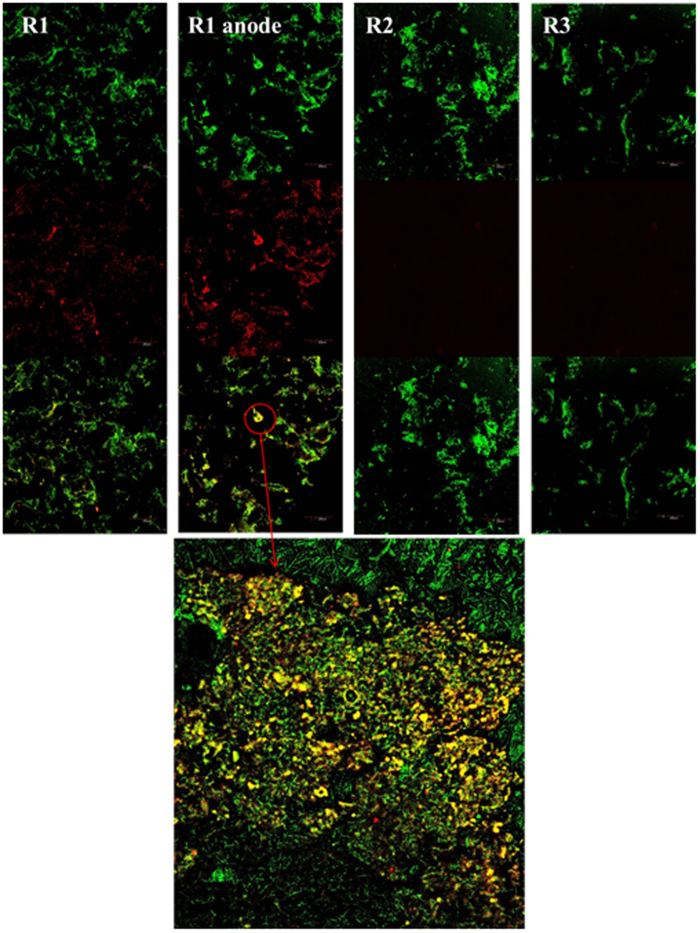
FISH images of the suspended sludge in R1, R2 and R3 and anodic biofilm of R1, respectively. The suspended sludge and anodic biofilm hybridized with specific probes for *Geobacter metallireducens* (GEO1-CY3, red) and *Methanosaeta* species (MX825-FITC, green).

Fluorescence *in situ* hybridization (FISH) was used to further demonstrate the potential DIET from *Geobacter* species and *Methanosaeta* species for methane production. The FISH analysis showed that the dominant *Geobacter* species enriched in the anodic biofilm and suspended sludge of R1 was just *Geobacter metallireducens* (GEO1-Cy3, AGAATCCAAGGACTCCGT, red) (Fig. 4), which had a relative abundance of 73.2% and 81.1% of total *Geobacter* species (GEO825-FITC, TACCCGCRACACCTAGT, green) *(Fig. S3)*, respectively. From FISH images, *Geobacter metallireducens* (ellipsoid, red) closely gathering with *Methanosaeta* species (long slender rods, green) implied the potential for biological interspecies electrical connections via DIET especially in the anodic biofilm of R1 (Fig. 4). Remarkably, there was almost no *Geobacter* species detected in the suspended sludge of R2 and R3 *(Fig. S3)*. This result was well in agreement with the bacterial community analysis via high-throughput 16S rRNA pyrosequencing.

## Discussion

Recently, DIET from *Geobacter* species to *Methanosaeta* species has been confirmed in defined co-culture of *G. metallireducens* and *M. harundinacea*^7^ as well as in brewery wastewater digesters^13^. Metatranscriptomic analysis revealed that the genes for CO_2_ reduction pathway in *M. harundinacea* were highly expressed, which caused that *Methanosaeta* species had the capacity to directly accept the electrons from *Geobacter* species for reduction of CO_2_ to CH_4_^35^. With the co-existence of *Geobacter* species and *Methanosaeta* species in an anaerobic digester, DIET is expected to be another important way to produce methane. *Geobacter* species is one of the most metabolically active microorganisms in the anaerobic environments, such as soils and sediments^17^, making DIET potential to contribute a considerable part of methane production in the world. However, the population of *Geobacter* species is pretty scare in waste activated sludge (Fig. 3B and Fig. 4), which makes DIET difficult to take place. Recently, some reported that the conductive carbon material, such as granular activated carbon (GAC)^36^, biochar^37^, carbon cloth^38^ and carbon nanotube^39^, were added into the methanogenic digesters to enhance conversion of wastes to methane via DIET. Differently, although a pair of graphite electrodes installed into the reactor (R3) also possibly serving as a similar conductive material to enhance the electron exchange in DIET, the increased methane production was insignificant (P > 0.05) as compared with the reactor with no electrodes (R2) (Fig. 1A). The lack of *Geobacter* species was the major reason limiting DIET for methane production during anaerobic sludge digestion.

With an electric supply imposed on the electrodes, although the changes of relative abundance of *Methanosaeta* species was not apparent, the enrichment of *Geobacter* species was obviously observed in the suspended sludge and especially in the anodic biofilm (Fig. 3). Bond and Lovley^24^ first revealed that electrode reduction could support the growth of *Geobacter* species. Further studies reported that *Geobacter* species usually adapt to grow with electrodes or Fe (III) oxides as electron acceptors^21^. In agreement, the electrodes with the power supply installed into the anaerobic digester created a favorable condition to enrich *Geobacter* species. Further FISH analysis showed that the dominant *Geobacter* species enriched in the anodic biofilm as well as in the suspended sludge of R1 was *Geobacter metallireducens*, which were well-known as the microorganism capable of DIET for methane production in defined co-cultures^7^,^10^ as well as in anaerobic methanogenic digesters^13^,^40^. Unlike other *Geobacter* species, *Geobacter metallireducens* not only utilize acetate as substrate for extracellular electron transfer but also utilize other SCFAs and alcohols^17^. It made *Geobacter metallireducens* more likely to grow in the anode of bioelectrochemical system fed with complex substrates as compared with other *Geobacter* species^41^,^42^,^43^,^44^. Another potential evidence to support this was that the current density of R1 dropped from day 18 to 30 (Fig. 1C). This was because acetate as the most favorite substrate for *Geobacter* species to produce electricity was almost depleted at day 15 *(Fig. S2)*. Afterwards, with enriching *Geobacter metallireducens*, it began to again utilize propionate or other SCFAs which allowed to recover the electricity production.

**Figure 5:**
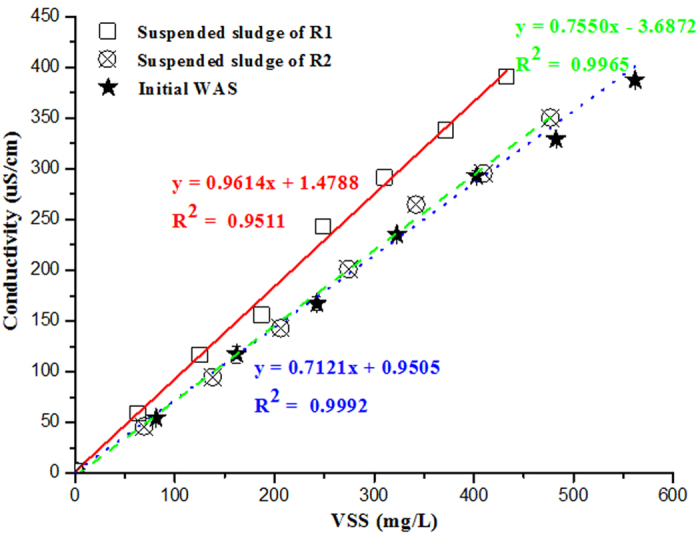
Conductivity (μS/cm) with the increase of VSS (mg/L) in the initial pretreated sludge and suspended sludge of R1 and R2 after 51 days experiments. Error bars represent standard deviations of three groups of parallel experiments.

The electrically conductive pili produced by *Geobacter* species for long-range electron exchange is the important mechanism for DIET^45^,^46^. If *Geobacter* species could exchange electron with methanogens through its conductive pili, the conductivity of sludge likely increased due to the participation of conductive pili^13^,^47^. The conductivity (μS/cm) in the suspended sludge before and after digestion presented a highly linear growth with the increase of VSS (mg/L) (Fig. 5). The average conductivity (slope of the curve, μS/cm/VSS) in the initial sludge (0.7121 ± 0.0025 μS/cm/VSS) and in the digested sludge of R2 (0.7550 ± 0.0045 μS/cm/VSS) were similar, both about thirty percentage points lower than that in the digested sludge of R1 (0.9614 ± 0.0079 μS/cm/VSS). The higher conductivity of the digested suspended sludge of R1 might be resulted from the direct interspecies electron exchange between the two species.

It is worth mentioning that bioelectrochemical methanogenesis in most of recent literatures was ascribed to the anodic oxidation of organics coupled with the cathodic reduction of CO_2_ into CH_4_^31^,^32^,^48^. Some considered that the more diverse communities formed on electrodes was a result for the increase of producing methane^49^. All of the present reports on bioelectrochemical methanogenesis have ignored the potential of DIET from *Geobacter* species to *Methanosaeta* species for methane production. Actually, the mechanism of anodic oxidation in the bioelectrochemical system was just that exoelectrogenic bacteria like *Geobacter* species transfer electrons from the oxidation of organic matters to electrodes. This study highly suggested that *Methanosaeta* species might be another sink to accept electron from *Geobacter* species in bioelectrochemical system.

**Figure 6:**
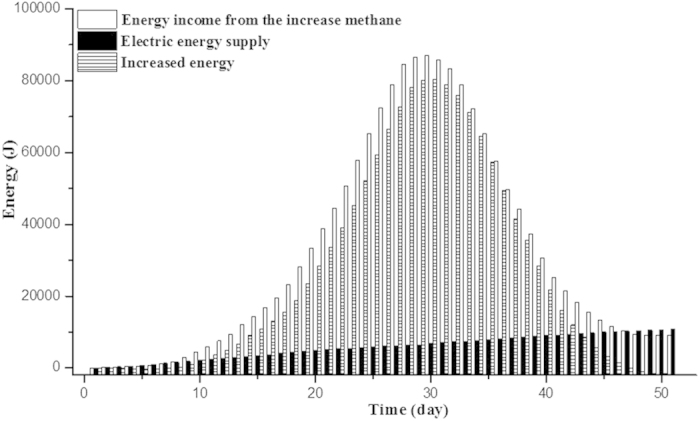
Change of energy in R1 during 51 days experiments.

Also, the electric energy supply for the electrodes inserted into is quite lower compared with the increased energy from methane production (Fig. 6). Energy income from the increased methane production (W_CH4_) of R1 (as compared with R2) reached 7.8 × 10^4^ J during the initial 33 days calculated by the formula (3), while the sum of electricity energy supply (W_E_) for the electrodes of R1 was only 7487.2 J calculated by the formula (2). It meant that the extra energy income was 10.5 folds (10.5 = 78757.4 J [W_CH4_] / 7487.2 J [W_E_]) of the electric energy supply during 33 days. Normally, the disconnection of the voltage supply in the bioelectrochemical system (opened R1) should be operated to further clarify the effects of DIET on methanogenesis. However, with *Geobacter* species gradually enriched in R1, the available substrates were progressively exhausted in this batch experiment. Assuming in the continuous feed mode, after the *Geobacter* species was enriched DIET was likely to continuously occur even shifting to the voltage-off state. It might obtain higher energy efficiency.

After 51 days experiments, the organic matter removal and sludge reduction are illustrated in Table S1 (see supplementary material). From this table, the effluent TSS, VSS and TCOD in R2 was 10320 ± 960 mg/L (mean ± standard deviation), 33500 ± 400 mg/L and 19007.2 ± 165.3 mg /L respectively, which was still similar to that in R3 (103150 ± 850 mg/L, 33600 ± 950 mg/L and 18940.5 ± 428.3 mg/L respectively). While, the effluent TSS, VSS and TCOD in R1 (96100 ± 700 mg/L, 31310 ± 700 mg/L and 16219.7 ± 256.0 mg/L) was lower than that in the other two reactors. It indicated organic matter removal and sludge reduction was enhanced with addition of a pair of electrodes in this study.

## Methods

### Waste activated sludge and anaerobic inoculum sludge

Waste activated sludge (WAS) used in this study was obtained from a secondary sedimentation tank of a local municipal wastewater treatment plant based on the activated sludge process in Dalian, China. The collected sludge was stored at 4 ^o^C before use. The main characteristics (mean ± standard deviation) of the WAS are as follows: pH 7.14 ± 0.02, total suspended solids (TSS) 100800 ± 200 mg/L, volatile suspended solids (VSS) 43100 ± 434 mg/L, total chemical oxygen demand (TCOD) 51453.5 ± 494.5 mg COD/L, soluble chemical oxygen demand (SCOD) 2903.6 ± 239.1 mg/L, total carbohydrate 1893.3 ± 16.5 mg COD/L, total protein 7217.6 ± 16.4 mg COD/L, total short-chain fatty acids (SCFAs) 1556.5 ± 161.7 mg COD/L.

Anaerobic sludge used as the inoculum for methane production was obtained from a waste sludge treatment plant in Dalian, China. Before the experiments, it was cultured in a batch anaerobic reactor, which had a working volume of 10 L (internal diameter of 200 mm and height of 320 mm). The reactor was operated in a room temperature (22.0 ± 2.0 ^o^C) with a hydrolytic retention time (HRT) of 24 h. Glucose were used as the substrate (COD: 1000 mg/L), and NH_4_Cl and KH_2_PO_4_ were used as nitrogen and phosphorus sources (at ratio of COD: N: P 200: 5: 1), respectively. The trace elements were added according to the following composition: 1 mL/L of a trace element solution containing Zn at 0.37 mmol/L, Mn at 2.5 mmol/L, Cu at 0.14 mmol/L, Co at 8.4  mmol/L, Ni at 0.25 mmol/L, H_3_BO_3_ at 0.8 mmol/L and EDTA at 3.4 mmol/L.

### Pretreating waste activated sludge at pH 10 for 8 days and mixing with anaerobic inoculum sludge

Before anaerobic inoculum sludge mixed with the initial WAS, the initial WAS was anaerobically pretreated at pH 10 for 8 days according to the method by Zhang *et al.*^50^. The experiment of pretreating WAS at pH 10 for 8 days was conducted in a cylindrical anaerobic reactor with a working volume of 5 L (internal diameter of 200 mm and height of 160 mm). The reactor was also operated in a room temperature (22.0 ± 2.0 ^o^C) equipped with mechanical stirrer at a speed of 80 rpm. The fermentation pH was maintained at 10.0 **±** 0.2 by 4 M sodium hydroxide (NaOH). After the 8 days pretreatment, the fermentation pH was adjusted to 7.0 using 4 M hydrochloric acid (HCl) and then mixed with the anaerobic inoculum sludge with a ratio of 9:1. The main characteristics of the pretreated WAS are as follows: TSS 104667 ± 580 mg/L, VSS 40667 ± 869 mg/L, TCOD 52307.5 ± 1067.5 mg/L, SCOD 13322.4 ± 512.4 mg/L, total carbohydrates 1893.3 ± 9.0 mg COD/L, solute carbohydrates 1014.8 ± 28.6 mg COD/L, total proteins 1389.3 ± 120.2 mg COD/L, solute proteins 991.2 ± 27.3 mg COD/L and total SCFAs 5446.1 ± 260.5 mg COD/L. The mixed sludge of 400 mL was added to each of reactors for anaerobic digestion.

### Batch experimental setup and experimental procedure

Batch experiments of anaerobic sludge digestion were conducted in a cylindrical reactor, each of which had a working volume of 500 mL (internal diameter of 80 mm and height of 100 mm). The graphite-brush anode (external diameter of 25 mm and height of 80 mm, surface areas is 17671 mm^2^) and the graphite-rob cathode (external diameter of 7 mm and height of 80 mm, surface areas 1759.2 mm^2^) with a distance of 50 mm were installed into to form a single-chamber bioelectrochemical system (hereafter referred to as R1). The DC power sources (Zhaoxin, RXN-305D, China) connected with the electrodes were used as the electric supply. Two control experiments were operated in this study. One was conducted in a same reactor as R1 but without electrodes (hereafter referred to as R2). The other was also conducted in a same reactor as R1 with the same electrodes but not connected with the DC power (hereafter referred to as R3). Another two groups of parallel experiments as same as R1, R2 and R3 were operated simultaneously. All the reactors equipped with a gas and sludge sampling port placed in a shaker at a speed of 140–150 rpm. All the batch experiments were operated at a temperature of 37.0 ± 2.0 ^o^C.

In order to assess the potential of DIET for methane production, the single-chamber bioelectrochemical reactor with applied voltage of 0.6 V (R1) and the two control reactors (R2 and R3) were operated continuously for 51 days. At the same time, three parallel control experiments were operated in the reactors as same as R1, R2 and R3 respectively (hereafter referred to as R4, R5 and R6) with addition of CH_3_F (3% [v/v], 99%) to inhibit aceticlastic methanogenesis. Remarkably, CH_3_F, as a specific inhibitor only for the metabolism of aceticlastic methanogenesis but allowing the operation of H_2_/CO_2_-dependent CH_4_ production, has been widely used to monitor the changes in carbon flow in methanogenic systems^51^,^52^,^53^,^54^. Also, another two groups of parallel experiments as same as R4, R5 and R6 were operated simultaneously. After 33 days experiments, the suspended sludge collected from the bottom of R1, R2 and R3 and anodic biofilm collected from the anodic surface of R1 were used to analyze the microbial community structure. Then, the suspended sludge of R1 and R2 was collected to measure the conductivity.

### Chemical analysis

Analysis of total suspended solid (TSS), volatile suspended solid (VSS) and chemical oxygen demand (COD) (include total COD and solute COD) were conducted in accordance with Standard Methods for the Examination of Water and Wastewater. Proteins (include total proteins and solute proteins) were measured with Lowry’s method^55^ using bovine serum albumin as a standard solution. Carbohydrates (include total carbohydrates and solute carbohydrates) were measured by phenol-sulfuric acid method^56^ using glucose as a standard solution. The concentrations of SCFAs were analyzed using a gas chromatograph (Tianmei, GC-7900P/FID, China) according to the report by Jiang *et al.*^57^. The equivalent relationship between COD and substrates are as follows: 1.5 g-COD/g protein, 1.06 g-COD/g carbohydrate, 1.07 g-COD/g acetate, 1.51 g-COD/g propionate, 1.82 g-COD/g butyrate and 2.04 g-COD/g valerate. Gas collected from all the reactors was used a gas collecting bag and the component was analyzed by another gas chromatograph (Tianmei, GC-7900P/TCD, China). The pH was recorded using a pH analyzer (Sartorius PB-20, Germany). The current was determined by measuring the voltage across a high-precision resistor (10Ω) using a multimeter/data acquisition system (Hongge, PCI-821H, China)^58^. The conductivity was measured by using a conductivity meter (Leici, DDS-307, China). Before the conductivity measured, the initial pretreated sludge and the suspended sludge of R1 and R2 were diluted according to the dilution ratio of 1:500, 2:500, 3:500, 4:500, 5:500, 6:500 and 7:500 respectively. Then the results measured were analyzed via linear fitting of Origin 8.0 and the slope (μS/cm/VSS) of the curve was the conductivity of the suspended sludge at room temperature (22.0 ± 2.0 ^o^C).

### DNA extraction, PCR amplification and high-throughput 16S rRNA pyrosequencing

After 51 days experiments, two types of sludge samples were collected to analyze the microbial community via high-throughput 16S rRNA gene pyrosequencing according to the method by Lu *et al.*^18^. One was the suspended sludge with the same volume (10 mL) taken from the bottom of R1, R2 and R3 and then harvested by centrifugation (110 × 100 g for 15 min at 4 °C). The other was the anodic biofilm collected from the anodic surface of R1 which was rinsed twice by phosphate-buffered saline (PBS; 0.13 M NaCl and 10 mM Na_2_HPO_4_ at pH 7.2) and then harvested by centrifugation (110 × 100 g for 15 min at 4 °C). The genomic DNA of the sludge samples were extracted using an extraction kit (Bioteke Corporation, Beijing, China) according to the manufacturer’s instructions. The quality of the extracted DNA was checked by determining its absorbance at 260 and 280 nm.

V1–V3 region (length of 455 bp) of the bacterial 16S rRNA gene was amplified using the universal primers 8F (5’-AGAGTTTGATCCTGGCTCAG-3’) and 533R (5’-TTACCGC GGCTGCTGGCA C-3’), as well as the 454 adapter A and B, barcode and linker sequence. After being purified and quantified, pyrosequencing was carried out by a Roche 454 FLX Titanium sequencer (Roche 454 Life Sciences, Branford, CT, USA) according to the standard protocols.

To analyze the microbial community of the two types of sludge samples, the sequences obtained were phylogenetically allocated down to the phylum, class and genus level using the MOTHUR program (http://www.mothur.org/wiki/Main) at a 0.03 distance level (97% similarity), and a confidence threshold of 95% was set for the phylogenetic classification. After phylogenetic allocation of the sequences down to the phylum, class and genus level, relative abundance of a given phylogenetic group was set as the number of sequences affiliated with that group divided by the total number of sequences per sample.

### Fluorescence ***in situ*** hybridization

Fluorescence ***in situ*** hybridization (FISH) was used to further demonstrate the potential DIET from *Geobacter* species to *Methanosaeta* species after 51 days experiments. The suspended sludge samples were taken from the bottom of R1, R2 and R3 with the same volume (5 mL), respectively and harvested by centrifugation (110 × 100 g for 15 min at 4 °C). The anodic biofilm of R1 were rinsed twice by phosphate-buffered saline (PBS; 0.13 M NaCl and 10 mM Na_2_HPO_4_ at pH 7.2) and harvested by centrifugation (110 × 100 g for 15 min at 4 °C). Three genus-specific probes for *Geobacter metallireducens* (GEO1-Cy3, AGAATCCAAGGACTCCGT, red)^10^, *Geobacter* species (GEO825-FITC, TACCCGCRACACCTAGT, green)^59^ and *Methanosaeta* species (MX825-FITC, TCGCACCGTGGCCGACACCTAGC, green)^7^ were used in this study. All the samples were rinsed another thrice by phosphate-buffered saline (PBS; 0.13 M NaCl and 10 mM Na_2_HPO_4_ at pH 7.2), and then fixed with 4% paraformaldehyde for 2 h at 4 °C. Hybridizations were performed at 46 °C for 1.5 h with buffer (0.9 M NaCl, 20 mM Tris-HCl [pH 7.2], 0.01 sodium dodecyl sulfate and 35% formamide) containing 50 ng probe per microliter and then washed with buffer (15 min at 48 °C)^31^. The samples were observed under a confocal laser scanning microscope (Leica SP2, Heidelberger, Germany). The FISH images were imported to Image-Pro Plus 6.0 for analysis of the relative abundance of microorganisms.

### Calculation

The amount of available electrons for cathodic reduction of CO_2_ to CH_4_ in R1 and R4 was evaluated by the following formula (1):
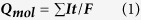
Where I is the average current (A/per day) calculated by current density (Fig. 1C), t is the time (24 × 3600 s/hr per day) and F is faradays constant (96485 C/mol). The amount of electric energy supply (W_E_ [J]) in R1 added to the circuit by DC power source, adjusted for loss across the resistor was calculated by the following equation (2)^34^:
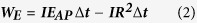
Where I is the average current (A/per day) based on current density (Fig. 1C), E_AP_ (V) is the applied voltage of R1 (0.6 V), ∆t (s) is the time of experiments (24 × 3600 s/hr per day), and R is the external resistor (10 Ω). The energy income of R1 from the increased methane production (W_CH4_ [J]) was calculated by the following equation (2):
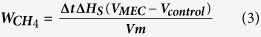
Where ∆Hs is the energy content of methane based on the heat of combustion (upper heating value, 890.31 × 10^3^ J/mol), ∆t (s) is the time of experiments (24 × 3600 s/hr per day), V_MEC_ is the volume of methane in R1 (L), V_Control_ is the volume of methane in R2 (L) and Vm is molar volume of the gas at room temperature and atmosphere pressure (24.8 L/mol).

## Additional Information

**How to cite this article**: Zhao, Z. *et al.* Potential for direct interspecies electron transfer in an electric-anaerobic system to increase methane production from sludge digestion. *Sci. Rep.* **5**, 11094; doi: 10.1038/srep11094 (2015).

## Supplementary Material

Supplementary Information
