# Supplementary material for: Potential for direct interspecies electron transfer in an electric-anaerobic system to increase methane production from sludge digestion
Source: Sci Rep. 2015 Jun 9;5:11094. doi: 10.1038/srep11094 (PMC4650609; doi:10.1038/srep11094)
Supplement: Supplementary Information [file srep11094-s1.doc]

Potential for direct interspecies electron transfer in an electric-anaerobic system to increase methane production from sludge digestion

**Authors**:

Zhiqiang Zhao^1^, Yaobin Zhang^1,2^ *, Liying Wang^2,3^, Xie Quan^1^

**Affiliations:**

^1^Key Laboratory of Industrial Ecology and Environmental Engineering (Dalian University of Technology), Ministry of Education, School of Environmental Science and Technology, Dalian University of Technology, Dalian 116024, China.

^2^Department of Microbiology, University of Massachusetts, Amherst, MA  01003-9298, USA

^3^State Key Laboratory of Bioreactor Engineering and Institute of Applied Chemistry, East China University of Science and Technology, Shanghai, P.R. China

*** Correspondence:** Tel: +86 411 8470 6460, Fax: +86 411 8470 6263;

E-mail address: zhangyb@dlut.edu.cn.

**Supporting Information: 4 pages, 1 table, 3 figures**

**Figure.S1.** Operational taxonomic units (OTUs) at a 3% distance detected in all the four archaeal microbial communities based on high-throughput 16S rRNA pyrosequencing.

**Figure.S2.** Change of effluent SCFAs in R1 during 51 days experiments.

**
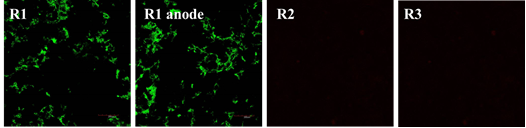
**

**Figure.S3.** FISH images of the suspended sludge in R1, R2 and R3 and anodic biofilm of R1, respectively. The suspended sludge and anodic biofilm hybridized with specific probes for total *Geobacter* species (GEO825-FITC, green).

**Table S1.** Performance of organic matter removal and sludge reduction after 51 days experiments. Error bars represent standard deviations (SD) of three groups of parallel experiments.

| **Item** | **Initial sludge** | **R1 effluent** | **R2 effluent** | **R3 effluent** |
| --- | --- | --- | --- | --- |
| TSS ± SD (mg/L) | 104667 ± 580 | 96100 ± 700 | 103200 ± 960 | 103150 ± 850 |
| VSS ± SD (mg/L) | 40667 ± 869 | 31310 ± 700 | 33500 ± 400 | 33600 ± 950 |
| TCOD ± SD (mg/L) | 52307.5 ± 1067.5 | 16219.7 ± 256.0 | 19007.2 ± 165.3 | 18940.5 ± 428.3 |
| pH ± SD | 7.14 **±** 0.02 | 8.18 ± 0.04 | 8.12 ± 0.02 | 8.15 ± 0.01 |
